# Diagnostic accuracy of fluorine-18 fluorodeoxyglucose positron emission tomography for suspected primary and postoperative pyogenic spondylitis

**DOI:** 10.1186/s13018-023-03507-z

**Published:** 2023-01-10

**Authors:** Qingyu Zhang, Haotian Feng, Jianmin Li, Rongjie Feng

**Affiliations:** 1 Department of Orthopedics, Shandong Provincial Hospital Affiliated to Shandong First Medical University, No.324, Road Jing Wu Wei Qi, Jinan, 250021 Shandong China; 2 Department of Orthopedics, Qilu Hospital, Cheeloo College of Medicine, Shandong University, Jinan, 250012 Shandong China

**Keywords:** Spondylitis, ^18^F-FDG PET, PET/CT, Meta-analysis

## Abstract

**Objective:**

Fluorine-18 fluorodeoxyglucose positron emission tomography (^18^F-FDG PET) and PET/CT have been suggested for confirming or excluding musculoskeletal infection but the diagnostic value of this tool for pyogenic spondylitis remains to be confirmed. This meta-analysis was performed to verify the accuracy of ^18^F-FDG PET and PET/CT in diagnosing suspected pyogenic spondylitis by performing a systematic review and meta-analysis.

**Methods:**

We conducted a comprehensive literature search of PubMed, Embase and Cochrane Library to retrieve diagnostic accuracy studies in which suspected pyogenic spondylitis was assessed with ^18^F-FDG PET or PET/CT. The pooled sensitivity, specificity, likelihood ratios, diagnostic odds ratio (DOR), summarized receiver operating characteristic curve (sROC) and the area under the sROC (AUC) were calculated by using Stata software.

**Results:**

A total of 18 eligible studies (660 patients) with suspected pyogenic spondylitis were included in the quantitative analysis. ^18^F-FDG PET and PET/CT illustrated relatively high sensitivity (0.91, 95% CI: 0.84–0.95) and specificity (0.90, 95% CI: 0.79–0.95) for the diagnosis of pyogenic spondylitis. The pooled DOR and AUC were 86.00 (95% CI, 31.00–240.00) and 0.96 (95% CI, 0.94–0.97), respectively. For diagnosing pyogenic spondylitis without previous spine surgery, the pooled sensitivity, specificity, DOR and AUC were 0.93 (95% CI, 0.85–0.97), 0.91 (95% CI, 0.77–0.97), 136 (95% CI, 35–530) and 0.97 (95% CI, 0.95–0.98), respectively. For diagnosing postoperative pyogenic spondylitis, the pooled sensitivity, specificity, DOR and AUC were 0.85 (95% CI, 0.71 to 0.93), 0.87 (95% CI, 0.66 to 0.96), 38 (95% CI, 9 to 167) and 0.92 (95% CI, 0.89 to 0.94), respectively.

**Conclusion:**

^18^F-FDG PET and PET/CT presented satisfactory accuracy for diagnosing pyogenic spondylitis. The diagnostic effect of this nuclear imaging method for pyogenic spondylitis without previous spine surgery seems to be better than that for the postoperative ones. However, whether ^18^F-FDG PET and PET/CT could become a routine in patients with suspected pyogenic spondylitis remains to be confirmed.

**Level of evidence:**

Level I evidence, a summary of meta-analysis.

**Supplementary Information:**

The online version contains supplementary material available at 10.1186/s13018-023-03507-z.

## Introduction

Pyogenic spondylitis refers to the infection of the spine, which encompasses a broad range of clinical entities (pyogenic spondylodiscitis, vertebral osteomyelitis and epidural abscess) [1, 2]. This devastating disorder accounts for 2–7% of all cases of musculoskeletal infection [2], and along with the aging of the population, the prevalence of pyogenic spondylitis is increasing [3, 4]. Meanwhile, postoperative spondylitis constitutes an important complication of spine surgery, which often leads to hospitalization/reoperation, increased morbidity and high economic burden [5]. Most patients with pyogenic spondylitis can achieve a satisfactory prognosis with conservative treatment at the early stage, but for patients with spinal cord or cauda equina compression and progressive neurological deficits, surgical intervention is indicated [5]. Early and accurate identification of pyogenic spondylitis is necessary to initiate timely management and improve the long-term outcome. However, the symptoms and signs of pyogenic spondylitis are often unspecific, diffuse and treacherous (e.g., fever, mild back pain), resulting in diagnostic difficulty.

A combination of clinical symptoms, laboratory tests and imaging investigations are necessary for assessing suspected pyogenic spondylitis in the clinic. The basic diagnostic examinations to establish a diagnosis of spondylitis include conventional X-ray, magnetic resonance imaging (MRI), computed tomography (CT), biopsy, blood culture and multiple nuclear imaging techniques. Changes in inflammatory markers such as C-reactive protein (CRP) and erythrocyte sedimentation rate (ESR) are evident on examination only after 4–8 weeks from the onset of the symptom [6, 7]. A substantial number of patients with chronic infection have normal or near-normal inflammatory markers [6]. Biopsy and blood culture are only successful in about half of the suspected patients to obtain a definite diagnosis and therefore are more suitable in the acute phase of spondylitis [8]. X-ray imaging and CT are relatively insensitive to diagnosing early spondylitis and predominantly used to guide biopsy [5]. Whereas MRI has high diagnostic accuracy for infection in the non-operated spine (sensitivity and specificity of > 90%) and therefore was ranked as the golden standard, detecting postoperative spondylitis by MRI remains challenging [9, 10]. Other shortcomings of MRI for assessing spondylitis are artifacts caused by metallic implants, similarities among pyogenic spondylitis, fracture and degenerative spinal diseases, and compromised sensitivity in patients with short-term symptoms [9, 10]. Therefore, additional practical tools are needed for diagnosing pyogenic spondylitis.

Fluorine-18 fluorodeoxyglucose positron emission tomography (^18^F-FDG PET) and PET/CT can be used to appraise increased glucose uptake and therefore are usually recommended to diagnose, stage, and detect the recurrence or progression of the malignant tumors [11]. Studies using this nuclear imaging technique to assess infection/inflammation are growing in recent years, especially for musculoskeletal infections [12]. Compared to CT and MRI, ^18^F-FDG PET and PET/CT provide the advantages of whole-body coverage to detect unintended metastasis of infection, and fewer artifacts due to metallic implants. Meanwhile, it can differentiate infections in bone or soft tissue. Thus, ^18^F-FDG PET and PET/CT have been suggested for assessing spinal infection when other imaging modalities fail to provide a definitive diagnosis or patients do not respond to antibiotic therapy as expected. A series of studies investigated the diagnostic accuracy of ^18^F-FDG PET and PET/CT for suspected spondylitis; however, these studies have relatively small sample sizes and limited power individually [3, 5, 9, 10]. Meanwhile, their reported results were controversial.

Several systematic reviews qualitatively appraised these studies but did not calculate the pooled diagnostic accuracy. A meta-analysis published in 2019 by Kim et al. further compared the diagnostic accuracy of ^18^F-FDG PET and MRI for spondylodiscitis [9]. However, this investigation only included seven studies published before 2018. Multiple large-scale studies specifically investigating the diagnostic efficiency of ^18^F-FDG PET for spondylitis were ignored. A literature search revealed no previous meta-analysis that provided a general overview of the ^18^F-FDG PET and PET/CT for diagnosing suspected spondylitis. With this in mind, this study aims to quantitatively clarify this issue by performing a systematic review and meta-analysis. Meanwhile, the diagnostic accuracy of this nuclear technique for suspected spondylitis with and without previous spine surgery was investigated separately.

## Materials and methods

The methodological approach to evidence searching and synthesis described in this article was following the Preferred Reporting Items for a Systematic Review and Meta-analysis of Diagnostic Test Accuracy Studies (PRISMA-DTA) statement [13]. Two investigators (QY Zhang and HT Feng) independently conducted electronic database searching, eligibility assessment, data extraction and methodological quality appraising. Any disagreement was settled through discussion and consensus. Informed consent and ethical approval were not needed because all data were retrieved from public databases.

### Search strategy

We systematically searched three electronic databases including PubMed, Embase, and Cochrane Library to retrieve entries published before March 15, 2022. Vocabulary and syntax were specifically adapted according to the database. We used (“spondylodiscitis” OR “discitis” OR “spondylitis” OR “vertebral osteomyelitis” OR “epidural abscess”) as the diagnosis of interest and (“fluorine-18 fluorodeoxyglucose positron emission tomography” OR “^18^F-FDG PET”) as the index tests. No language limitation or other search filters were applied. Bibliographies of related articles were also screened manually to identify additional eligible records.

### Inclusion and exclusion criteria

Studies eligible for this meta-analysis needed to meet all the following criteria: (1) study design, diagnostic accuracy study, (2) population, patients with suspected pyogenic spondylitis, (3) ^18^F-FDG PET or PET/CT was performed, (4) the final diagnosis of pyogenic spondylitis was confirmed by predesigned reference criteria; and (5) enough data could be extracted to construct a 2 × 2 contingency table to determine the diagnostic performance of index test.

Exclusion criteria were (1) case–control studies; (2) studies not specifically investigating suspected pyogenic spondylitis; (3) studies with fewer than five participants; and (4) expert opinions, comments, letters and editorials.

The titles and abstracts were first assessed to exclude apparently ineligible studies, and the final decision on inclusion was based on downloading and scrutinizing the full articles. For studies with overlapping participants, the latest one was included. Inter-reviewer agreement was assessed at each stage by using the Kappa (κ) statistic: a *k* > 0.6, substantial agreement, a *k* between 0.21 and 0.6, moderate agreement, and a *k* < 0.2, poor agreement.

### Data extraction

The following information was extracted and recorded into a standardized excel file: first author’s last name, year and region of publication, study design, inclusion interval of participants, number and demographic information of participants, standardized references, number of patients with suspected spondylitis, type and methodological data of applied nuclear imaging modality, history of spine surgery, presence or absence of implants, as well as the raw diagnostic data (number of false/true-positive [FP/TP] and false/true-negative [FN/TN] cases).

### Quality assessment

The methodological quality of included studies was appraised according to the QUADAS (Quality Assessment of Diagnostic Accuracy Studies)-2 tool [14], which consisted of eleven questions. These questions were answered with “yes” for a low risk of bias/concerns, “no” for a high risk of bias/concerns or “unclear” when the relevant information was not clearly provided.

### Statistical analyses

The pooled positive incidence was computed by generating the proportion of the yield (true-positive) and associated 95% confidence intervals (CI) using a random effect model. For the diagnostic modalities, TP, FP, TN and FN results were derived from a two-by-two contingency table. The pooled sensitivity, specificity, positive likelihood ratio (PLR), negative likelihood ratio (NLR), diagnostic odds ratio (DOR), summarized receiver operating characteristic curve (sROC) and the area under sROC (AUC) were constructed utilizing the original diagnostic data. Heterogeneity across included studies was assessed using the *I*^2^ statistic. An *I*^2^ value of 0% implied no heterogeneity, and values of > 50% indicated substantial heterogeneity. Publication bias was assessed using Deeks' Funnel Plot Asymmetry. All statistical analyses were finished using STATA (Version 16.0, StataCorp, College Station, TX).

## Results

### Included studies

**Fig. 1:**
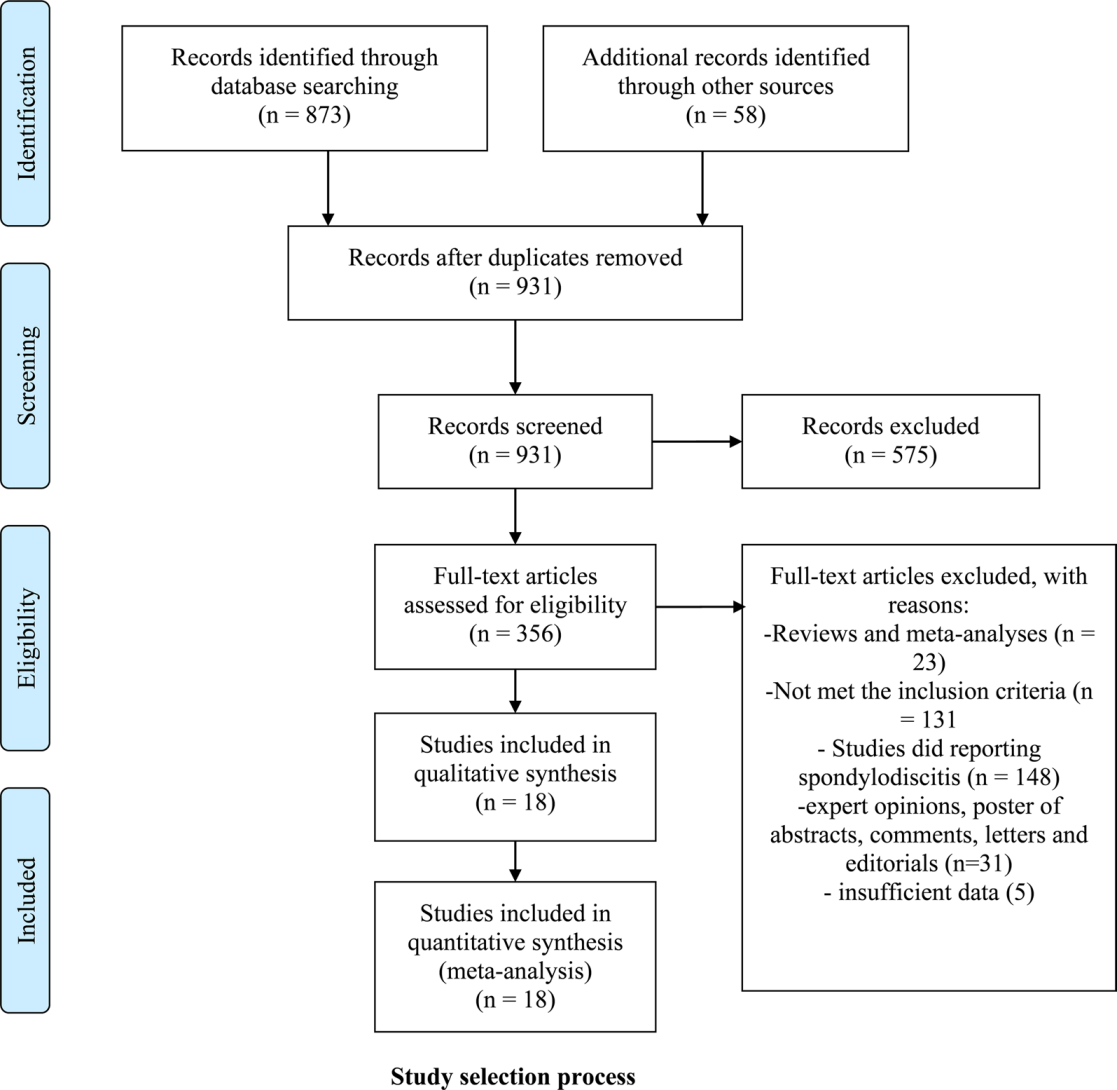
Selection process of included studies

A total of 931 unique records were identified by searching electronic databases and screening reference lists of relevant articles. After the initial screening of titles and abstracts, 356 articles were further assessed by scrutinizing the full texts against the predesigned criteria, and the eligibility of each article was established by a group discussion until consensus was reached. Eventually, 18 [5–7, 15–29] articles published during the period from 2001 to 2021 were selected for quantitative analysis. Selection processes for eligible studies are summarized in Fig. 1. There was a substantial inter-reviewer agreement as to the title (*κ* = 0.94) abstract (*κ* = 0.87) and full-text screening (*κ* = 0.97) stages.

### Study characteristics

**Fig. 2:**
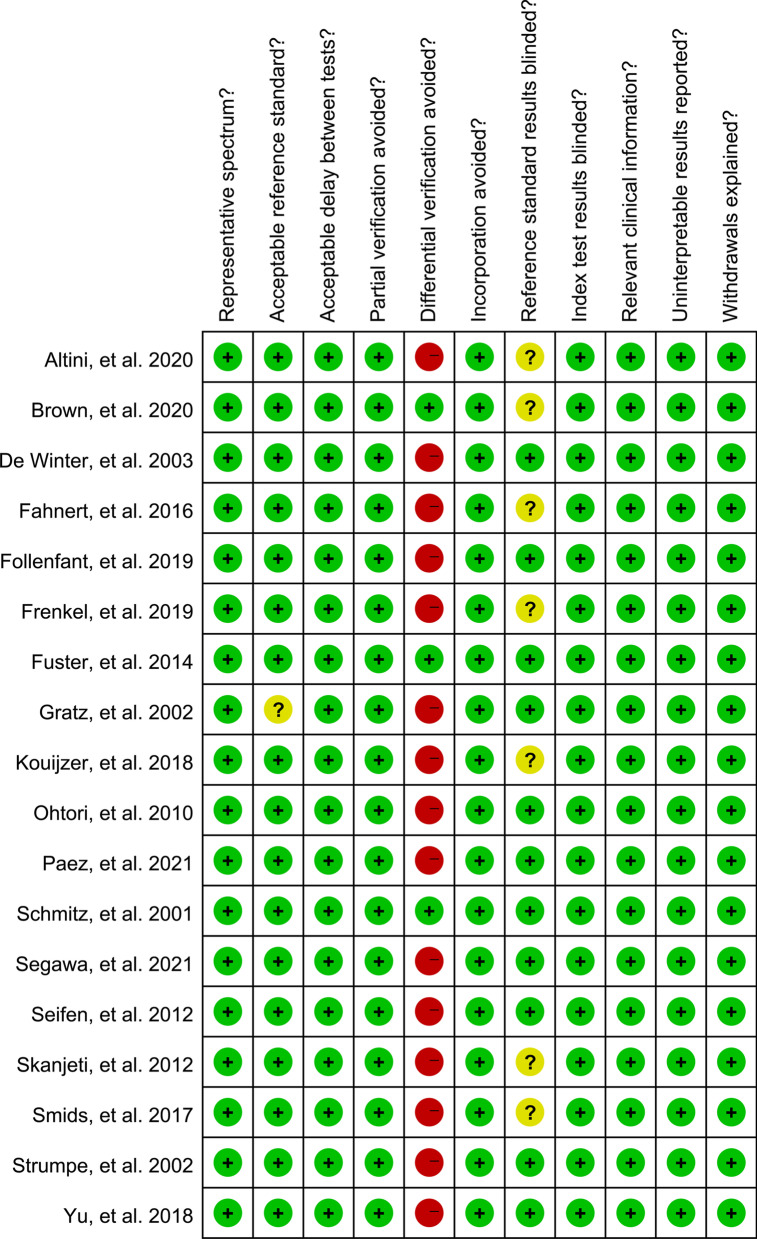
Methodological quality summary of the included studies. Red indicates a high risk of bias, yellow an unclear risk of bias and green a low risk of bias

The sample size of included studies ranged from 9 to 149 with a total of 660 patients, of which 332 ones were identified with pyogenic spondylitis. Nine [5, 18, 19, 21, 22, 25–28] studies used prospective study design, whereas nine [6, 7, 15–17, 20, 23, 24, 29] were retrospective ones. Twelve [5, 7, 15, 19, 21–23, 25–29] studies claimed that the nuclear medicine specialists were blinded to the final diagnosis of reference tests, while six [5, 15, 18, 20, 24, 29] studies did not clarify this fact. Among included studies, seven [21, 24–29] focused on ^18^F-FDG PET and 11 [5–7, 15–20, 22, 23] used ^18^F-FDG PET/CT. A schematic overview of the included studies is presented in Table 1, and the technical details are listed in Table 2. As for the methodological quality of included studies, fifteen studies were identified to have high risk of bias in the term “differentially verification avoided” because the reference standard was not uniform (Fig. 2).

**Table 1:** Main characteristics of the included studies

Author	Year	Region	Design	Blind (= 0 if NR, = 1 if Y)	Inclusion interval	Number of patients	Patients’ demographics (male/female)	Age, yrs	Type of infection	Number of infected patients
Paez, et al.	2021	Austria	P	1	2013.12–2017.12	44	NA	NA	Postoperative spondylodiscitis	30
Segawa, et al.	2021	Japan	R	1	2008.12–2018.07	50	12/38	65.5 ± 17.4	Postoperative spondylitis	29
Brown, et al.	2020	UK	R	0	2012–2017	49	16/33	33(17–70)	Postoperative spondylitis	24
Altini, et al.	2020	Italy	R	0	2013.04–2018.10	56	39/17	18–90	Spondylodiscitis	48
Frenkel, et al.	2019	Israel	R	0	2011.01.01–2013.12.31	9	5/4	59.2 ± 16.4	Postoperative hardware-related spondylitis	5
Follenfant, et al.	2019	France	R	1	2010.4–2018.7	44	22/22	25–75	Postoperative hardware-related spondylitis	22
Kouijzer, et al.	2018	Netherlands	P	0	2015.11–2016.12	32	22/10	66.8(43–92)	Vertebral osteomyelitis	20
Yu, et al.	2018	Canada	P	1	2011–2015	149	94/55	NA	Pyogenic spondylitis	43
Smids, et al.	2017	Netherlands	R	0	2006.01–2013.08	68	35/33	NA	Spondylodiscitis	49
Fahnert, et al.	2016	Germany	P	1	2014.03–2015.08	28	10/18	NA	Spondylodiscitis	12
Fuster, et al.	2015	Spain	P	1	2008.12–2012.02	26	14/12	59 ± 17	Spondylodiscitis	18
Skanjeti, et al.	2012	Italy	R	0	2005.05–2010.04	24	13/11	56.9 ± 17.8	Spondylodiscitis	14
Seifen, et al.	2012	Austra	R	1	2006.05–2010.10	38	20/18	67 ± 14	Spondylodiscitis	22
Ohtori, et al.	2010	Japan	P	1	NR	18	14/4	66 ± 10.6	Pyogenic spondylitis	11
De Winter, et al.	2003	Belgium	P	1	1999.02–2001.06	57	27/30	13–74	Postoperative spondylitis	15
Strumpe, et al.	2002	Switherland	P	1	2000.05–2000.10	30	11/19	54(27–80)	Spondylodiscitis	5
Gratz, et al.	2002	Germany	P	1	1999.06–2000.12	16	9/7	59 (25–78)	Spondylitis	12
Schimitz, et al.	2001	Germany	R	1	NR	16	10/6	15–85	Spondylodiscitis	12

*R* retrospective; *P* prospective; *NA* not available; *NR* not reported; *Y* yes

**Table 2:** Technical aspects of the included studies

Author	Year	Reference standard	Type of nuclear medicine methods	Injected dose (MBp)	Time between injection and image acquisition	Threshold
Paez, et al.	2021	biopsy or follow-up for at least 6 months	PET/CT	NA	NA	Score 3 and 4
Segawa, et al.	2021	biology or clinical observation	PET/CT	up to 370 MBq	60 min	5.0 for SUVmax
Brown, et al.	2020	microbiology assessment	PET/CT	200 MBq	60 min	NA
Altini, et al.	2020	resolution or significant improvement of constitutional symptoms	PET/CT	2.5–3 MBq/kg	60 min	NA
Frenkel, et al.	2019	clinical and bacteriological findings	PET/CT	296–555 MBq	60–90 min	NA
Follenfant, et al.	2019	observation of pus surrounding the implant during a new surgery and/or the identification of a microorganism from cultures of bone tissue or blood	PET/CT	NA	60 min	NA
Kouijzer, et al.	2018	IDSA guideline, imaging and clinical findings	PET/CT	3.3 MBq/kg	60 min	NA
Yu, et al.	2018	bacterial cultures and outcomes after cessation or withholding of antibiotic therapy	PET/CT	550 MBq	60 min	NA
Smids, et al.	2017	a microorganism was isolated and/or imaging follow-up showing response to antibiotic therapy	PET/CT	200 MBq	60 min	NA
Fahnert, et al.	2016	histopathologic evaluation of surgery or biopsy specimens or clinical follow-up	PET	4 MBq/kg; range, 149–410 MBq	75 min (60–105 min)	SUVmax ratio of 2.1
Fuster, et al.	2015	micrological documentation in cultures of image-guided spinal puncture fluid or blood	PET/CT	4.07 MBq/kg	60 min	SUVmax ratio of 2.2
Skanjeti, et al.	2012	a long diagnostic work-up including spinal MRI, blood cultures or vertebral biopsy	PET	222–370 MBq	60 min	NA
Seifen, et al.	2012	biopsy, blood culture and a one-year clinical follow-up	PET/CT	4 MBq/kg; range, 253–493 MBq	60 min	NA
Ohtori, et al.	2010	symptoms, x-ray imaging, MRI, blood data, and results of biopsies from the first examination to final follow-up	PET	NA	NA	NA
De Winter, et al.	2003	histopathology, microbiology and/or intraoperative visual assessment or minimum follow-up of 12 months	PET	370 MBq	60–90 min	NA
Strumpe, et al.	2002	cultures from the bone biopsies or blood, clinical and laboratory findings	PET	300–400 MBq	30–40 min	NA
Gratz, et al.	2002	biopsy, histology, culture, clinical and laboratory findings	PET	296 MBq	60 min	NA
Schimitz, et al.	2001	histopathological examination	PET	200–400 MBq	45 min	NA

*R* retrospective; *P* prospective; *NA* not available; *IDSA* Infectious Diseases Society of America

### Meta-analysis

#### Incidence of a positive result

**Fig. 3:**
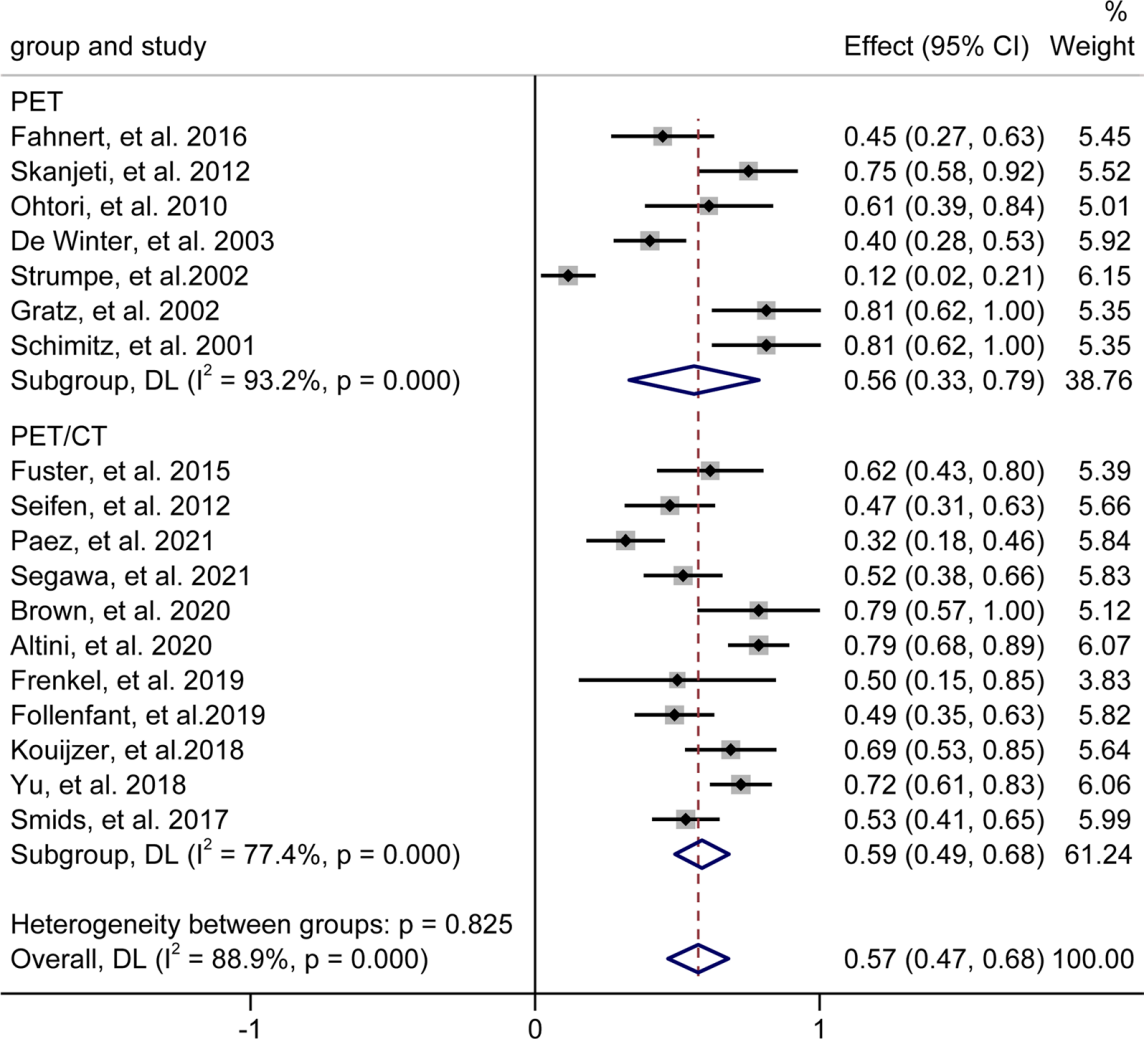
Overall incidence of a positive result of 18F-FDG PET and PET/CT in patients with suspected pyogenic spondylitis

The overall incidence of positivity of ^18^F-FDG PET as generated from seven [21, 24–29] datasets was 56% (95% CI: 33–79%) for suspected pyogenic spondylitis (Fig. 3). For ^18^F-FDG PET/CT, the overall incidence of positivity as generated from 11 [5–7, 15–20, 22, 23] datasets was 59% (95% CI: 49–68%), demonstrating a similarly positive result with ^18^F-FDG PET (Fig. 3).

#### Overall diagnostic value of ^18^F-FDG PET or PET/CT for pyogenic spondylitis

**Fig. 4:**
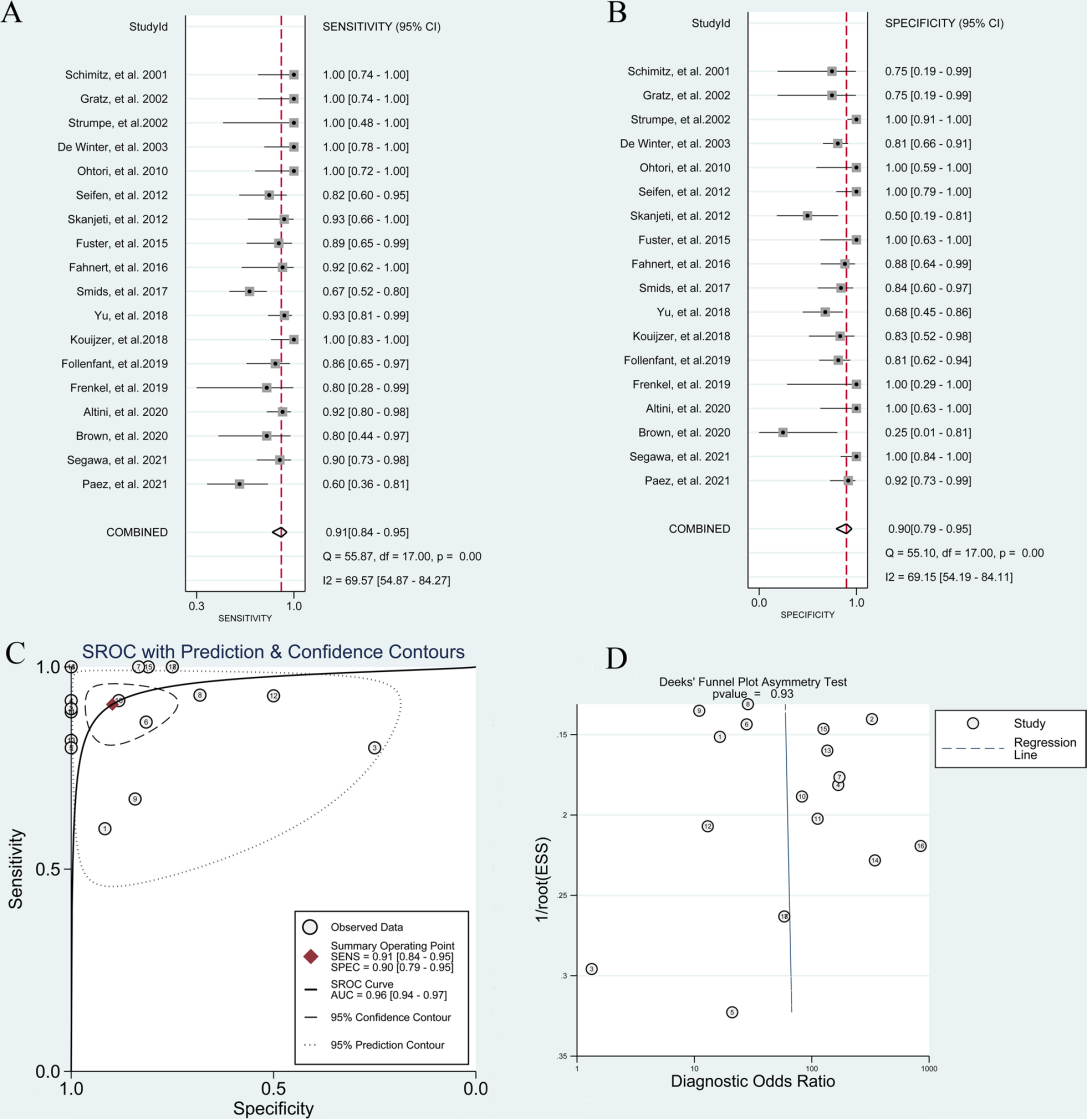
Diagnostic performance of ^18^F-FDG PET and PET/CT for pyogenic spondylitis: **A** pooled sensitivity **B** pooled specificity **C** summary receiver operating characteristic curve (sROC) with the Q*-index and **D** publication bias

The pooled sensitivity and specificity of ^18^F-FDG PET or PET/CT for diagnosing pyogenic spondylitis were 0.91 (95% CI, 0.84 to 0.95) and 0.90 (95% CI, 0.79 to 0.95), respectively (Fig. 4A and B). The pooled PLR, NLR, DOR and AUC were 8.9 (95% CI, 4.2 to 18.9), 0.10 (95% CI, 0.06 to 0.18), 86.00 (95% CI, 31.00 to 240.00) and 0.96 (95% CI, 0.94 to 0.97), respectively (Fig. 4C). Deeks' funnel plot asymmetry test revealed no existences of publication bias (*p* = 0.93, Fig. 4D).

#### ^18^F-FDG PET for pyogenic spondylitis

The pooled sensitivity and specificity of ^18^F-FDG PET for diagnosing pyogenic spondylitis were 0.98 (95% CI, 0.88 to 1.00) and 0.88 (95% CI, 0.69 to 0.96), respectively (Additional file 1: Figure S1A and B). The pooled PLR, NLR, DOR and AUC were 8.5 (95% CI, 2.8 to 26.1), 0.02 (95% CI, 0.00 to 0.16), 414 (95% CI, 30 to 5800) and 0.99 (95% CI, 0.98 to 1.00), respectively (Additional file 1: Figure S1C). No publication bias was identified, either (*p* = 0.93, Additional file 1: Figure S1D).

#### ^18^F-FDG PET/CT for pyogenic spondylitis

The pooled sensitivity and specificity of ^18^F-FDG PET/CT for diagnosing pyogenic spondylitis were 0.86 (95% CI, 0.78 to 0.91) and 0.91 (95% CI, 0.76 to 0.97), respectively (Additional file 2 and 1: Figures S2A and S1B). The pooled PLR, NLR, DOR and AUC were 9.6 (95% CI, 3.2 to 28.4), 0.16 (95% CI, 0.09 to 0.25), 62 (95% CI, 17 to 231) and 0.97 (95% CI, 0.95 to 0.98), respectively (Additional file 2: Figure S2C). Deeks' funnel plot asymmetry test did not reveal publication bias (*p* = 0.71, Additional file 2: Figure S2D).

#### ^18^F-FDG PET or PET/CT for pyogenic spondylitis without previous spine surgery

**Fig. 5:**
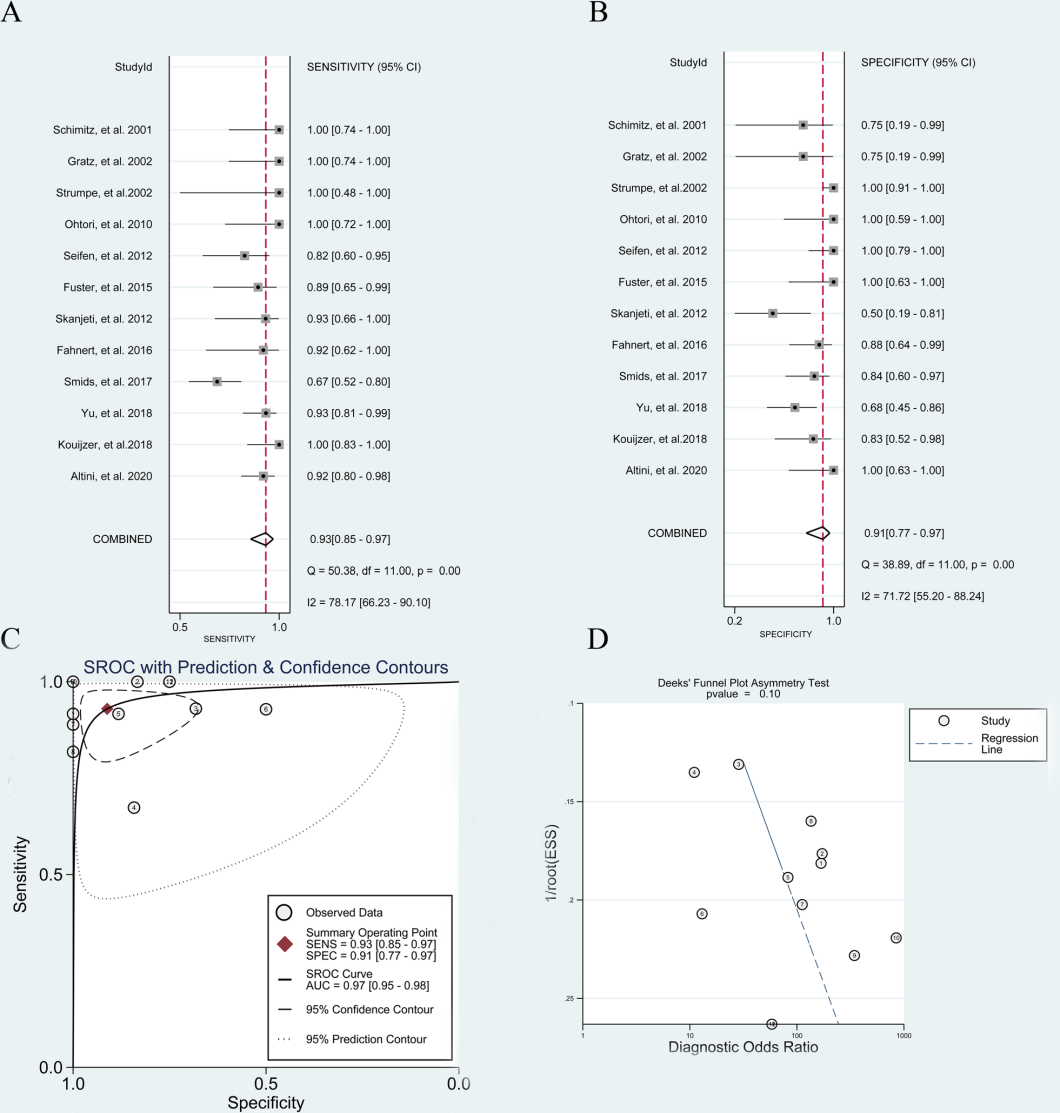
Diagnostic performance of ^18^F-FDG PET and PET/CT for spondylitis without spine surgery: **A** pooled sensitivity **B** pooled specificity **C** summary receiver operating characteristic curve (sROC) with the Q*-index and **D** publication bias

Twelve [16, 18–25, 27–29] studies investigated the accuracy of ^18^F-FDG PET or PET/CT for diagnosing pyogenic spondylitis without previous spine surgery. The pooled sensitivity and specificity were 0.93 (95% CI, 0.85 to 0.97) and 0.91 (95% CI, 0.77 to 0.97), respectively (Fig. 5A and B). The pooled PLR, NLR, DOR and AUC were 10.5 (95% CI, 3.90 to 28.60), 0.08 (95% CI, 0.04 to 0.17), 136 (95% CI, 35 to 530) and 0.97 (95% CI, 0.95 to 0.98), respectively (Fig. 5C). No publication bias was identified (*p* = 0.10, Fig. 5D).

#### ^18^F-FDG PET or PET/CT for postoperative spondylitis

**Fig. 6:**
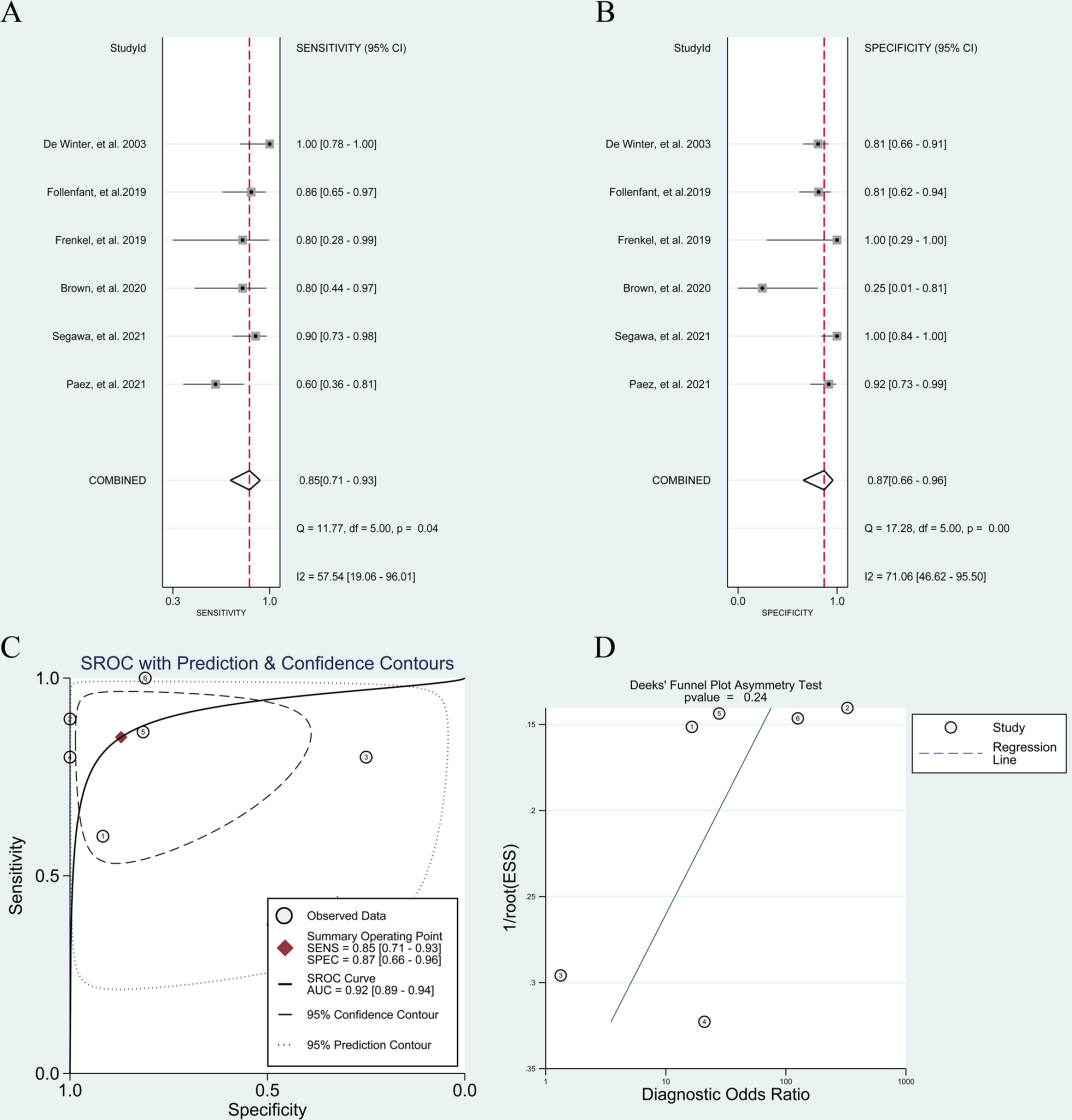
Diagnostic performance of ^18^F-FDG PET and PET/CT for postoperative spondylitis: **A** pooled sensitivity **B** pooled specificity **C** summary receiver operating characteristic curve (sROC) with the Q*-index and **D** publication bias

Six [5–7, 15, 17, 26] studies investigated the accuracy of ^18^F-FDG PET for diagnosing postoperative spondylitis. The pooled sensitivity and specificity were 0.85 (95% CI, 0.71 to 0.93) and 0.87 (95% CI, 0.66 to 0.96), respectively (Fig. 6A and B). The pooled PLR, NLR, DOR and AUC were 6.60 (95% CI, 2.20 to 19.4), 0.17 (95% CI, 0.08 to 0.36), 38 (95% CI, 9 to 167) and 0.92 (95% CI, 0.89 to 0.94), respectively (Fig. 6C). Deeks' funnel plot asymmetry test revealed no existence of publication bias (*p* = 0.24) (Fig. 6D).

### Qualitative analysis

#### Cut-off value of SUVmax values for the diagnosis of pyogenic spondylitis

Three [15, 21, 22] studies provided the cut-off values of ^18^F-FDG uptake for the diagnosis of spondylitis. Segawa et al. [15] revealed that all patients with a cut-off SUVmax of ≥ 5.0 had infection (specificity = 100%) and the sensitivity is 90%. Another study [22] applied the SUVmax of 5.324 as the threshold value, however, the sensitivity is only 56%. Fahner et al. [21] and Fuster et al. [22] adapted SUVmax ratios (SUVmax corrected by normal tissue) of 2.1 and 2.2, respectively. The reported specificities were 100% and 88%, respectively.

#### Presence of implants for the diagnostic yield

Studies reported by Frenkel et al. [17] and Follenfant et al. [7] only investigated the postoperative spondylitis with implants. These two studies found similar diagnostic values to the pooled performance of ^18^F-FDG PET and PET/CT for postoperative spinal infections. ^18^F-FDG PET and PET/CT could effect tools to narrow the surgical field for a successful revision surgery.

## Discussion

As noninvasive modalities, nuclear imaging techniques have become increasingly important for diagnosing infection/inflammation by identifying elevated glycolysis. Multiple studies specifically investigated the diagnostic accuracy of ^18^F-FDG PET or PET/CT for pyogenic spondylitis but no definitive result was obtained. To address this question scientifically, an updated pairwise meta-analysis was performed and the pooled results demonstrated that ^18^F-FDG PET and PET/CT had satisfactory accuracy (sensitivity = 0.91, 95% CI: 0.84–0.95; specificity = 0.90, 95% CI: 0.79–0.95) in identifying or excluding suspected pyogenic spondylitis. These results are partly consistent with the previously reported accuracy of this imaging technique for diagnosing suspected fracture-related infection and periprosthetic joint infection [12].

Currently, MRI is the most commonly used diagnostic procedure for pyogenic spondylitis. The manifestations include intervertebral disk injury, subsequent intervertebral stenosis, blurring of the endplates, height loss of the invaded vertebral bodies, possible epidural involvement and increased contrast enhancement in the spine [30, 31]. However, MRI is not a suitable examination for all patients. First, clinical evaluation by MRI is negatively affected by embedded implants due to the existence of artifacts [32]. Metallic hardware could induce perturbations to the static *B*_0_ magnetic field, disrupting the spatial encoding mechanism used in conventional MRI. Besides, for patients susceptible to multiple infections, whole-spine or whole-body examination using MRI needs high costs and a long run time. Last but not least, MRI cannot be performed in patients with implantable cardiac electronic devices, cochlear implants or other foreign metallic bodies [33]. Higher SUVs of ^18^F-FDG reflect an elevated risk for pyogenic spondylitis, providing valuable information to complement the morphological and structural data provided by MRI and CT [34]. Considering the high diagnostic accuracy, ^18^F-FDG PET and PET/CT can overcome the shortcoming of MRI mentioned above, fit for patients with internal implants or with suspected multiple infections. Meanwhile, compared with other alternative nuclear imaging methods (for instance, SPECT/CT), ^18^F-FDG PET and PET/CT are convenient and cost-effective by preventing unnecessary examinations and reducing duration of hospitalization, widely used in clinical practice [35].

Only three studies defined specific cut-off values of maximum standardized uptake value (SUVmax) for positivity [15, 21, 22]. From these results, a constant value of SUVmax to distinguish pyogenic spondylitis from non-infected cases is not realistic. A SUVmax ratio based on normal tissue is a preferable choice. Establishing criteria for identifying pyogenic spondylitis according to the dose of ^18^F-FDG administrated, and the characteristic of each center is imperative. In other studies, pattern-based diagnosis is employed. Through the joint application of semi-quantitative data (SUV) and qualitative assessment (distribution patterns and grades), ^18^F-FDG PET and PET/CT revealed considerable sensitivity in the diagnosis and appraisal of spondylitis foci throughout the body.

Postoperative spondylitis is a severe complication after spine surgery with and without instruments. The reported incidence varies from 1 to 15% with posterior surgery having a higher rate than anterior one [5, 6, 15]. It could be noticed that the most commonly cultured organism in spondylitis was *Mycobacterium tuberculosis* [5–7, 15, 17]. This infection could lead to osteolysis and the subsequent loosening of orthopedic implants [36]. Besides additional surgery and pain, postoperative spondylitis is associated with a high financial cost, averaging about 38, 000 dollars per patient [5]. Although not influenced by artifacts, ^18^F-FDG uptake will also be increased in postoperative patients without infection due to the local inflammation. In this study, we noticed that ^18^F-FDG PET and PET/CT revealed excellent sensitivity (0.85, 95% CI, 0.71–0.93) and specificity (0.87, (95% CI, 0.66 to 0.96) for diagnosing postoperative spondylitis, which seems to be lower than the diagnostic effect of ^18^F-FDG PET and PET/CT for pyogenic spondylitis without previous spine surgery. Further appraising using DOR, a parameter compromising the sensitivity and specificity, confirmed this conclusion.

Depending on the clinical scenario where ^18^F-FDG PET or PET/CT is employed, one might want to maximize the sensitivity to allow the scan to effectively rule out infection or to enhance specificity to ascertain the presence of pyogenic spondylitis. The reasons for the misdiagnoses are multifactorial. Increased ^18^F-FDG uptake is not pathognomonic for infectious diseases. Some benign lesions such as sterile inflammatory disease, granulomatous tissue and fractures also exhibit high levels of tracer accumulation. Therefore, the findings of ^18^F-FDG PET and PET/CT should be finally confirmed by histopathology examination or follow-up. Meanwhile, false-negative cases are inevitable. The first cause is the nonspecific ^18^F-FDG uptake and asymmetric ^18^F-FDG distribution in infectious diseases. Second, since the limited spatial resolution of ^18^F-FDG PET, occult or small lesions could not be identified. Third, some false-negative results are related to low-virulent bacteria.

Although this meta-analysis only investigated the diagnostic value of ^18^F-FDG PET and PET-CT, in comparison with the former two meta-analyses [9, 10], this study has three strengths. First, this investigation directly assessed the diagnostic accuracies of ^18^F-FDG PET and PET/CT in suspected spondylitis using a pairwise meta-analysis. Unlike the recent review [9] that only retrieved seven studies and focused on the comparison of ^18^F-FDG PET and MRI, we performed a thorough literature search and eventually eighteen studies were included. This is the most comprehensive analysis on this topic. Second, a subgroup meta-analysis was performed according to the type of pyogenic spondylitis, and main diagnostic parameters were obtained in each analysis. Distinct characteristics existed between spondylitis with spine surgery and those without surgical history, and in this study, these two groups of entities were analyzed separately. Last but not least, multiple statistical indicators as well as qualitative analysis were used to appraise the performance of ^18^F-FDG PET and PET/CT for spondylitis.

This study is not without limitations. A general shortcoming of included studies is the lack of a uniform reference standard for identifying spondylitis. We could not restrict inclusion to studies using biopsy or microbiological examination because these techniques only revealed limited sensitivity. Therefore, clinical follow-up sometimes was imperative to reconfirm the final diagnosis. Second, evidence of heterogeneity in diagnostic data existed throughout enrolled articles. Another major limitation is that subgroup analyses were not conducted during data merging based on essential variables such as whether metallic implants were used. Other minor shortcomings also merit consideration. For instance, the number of eligible studies is relatively small, and whether the results of nuclear imaging modalities were interpreted with blinding to the findings of reference test was not mentioned in nine studies, which could reduce the reliability of the result.

## Conclusions

Based on the results of the current meta-analysis, ^18^F-FDG PET or PET/CT presents satisfactory accuracy for the diagnosis of pyogenic spondylitis. The diagnostic effect of this nuclear imaging method for spondylitis without previous spine surgery seems to be better than that for the postoperative ones. However, whether ^18^F-FDG PET or PET/CT could become a routine in patients with suspected spondylitis remains to be confirmed.

## Supplementary Information

**Additional file 1: Fig. S1.** Diagnostic performance of ^18^F-FDG PET for pyogenic spondylitis: (A) pooled sensitivity (B) pooled specificity (C) summary receiver operating characteristic curve (sROC) with the Q*-index and (D) publication bias.

**Additional file 2: Fig. S2.** Diagnostic performance of ^18^F-FDG PET/CT for pyogenic spondylitis: (A) pooled sensitivity (B) pooled specificity (C) summary receiver operating characteristic curve (sROC) with the Q*-index and (D) publication bias.

## Data Availability

All data analyzed during this study are included in this published article.

## Abbreviations

CT: Computed tomography
MRI: Magnetic resonance imaging
18F-FDG: 18F-fluorodeoxyglucose
PET: Positron emission tomography
SUVmax: Maximum standardized uptake value
TP: True-positive
FP: False-positive
FN: False-negative
TN: True-negative
PLR: Positive likelihood ratio
NLR: Negative likelihood ratio
DOR: Diagnostic odds ratio
sROC: Summarized receiver operating characteristic
AUC: Area under curve
CI: Confidence interval
