# Supplementary figures and images for: Diagnostic accuracy of fluorine-18 fluorodeoxyglucose positron emission tomography for suspected primary and postoperative pyogenic spondylitis
Source: J Orthop Surg Res. 2023 Jan 10;18:23. doi: 10.1186/s13018-023-03507-z (PMC9830889; doi:10.1186/s13018-023-03507-z)

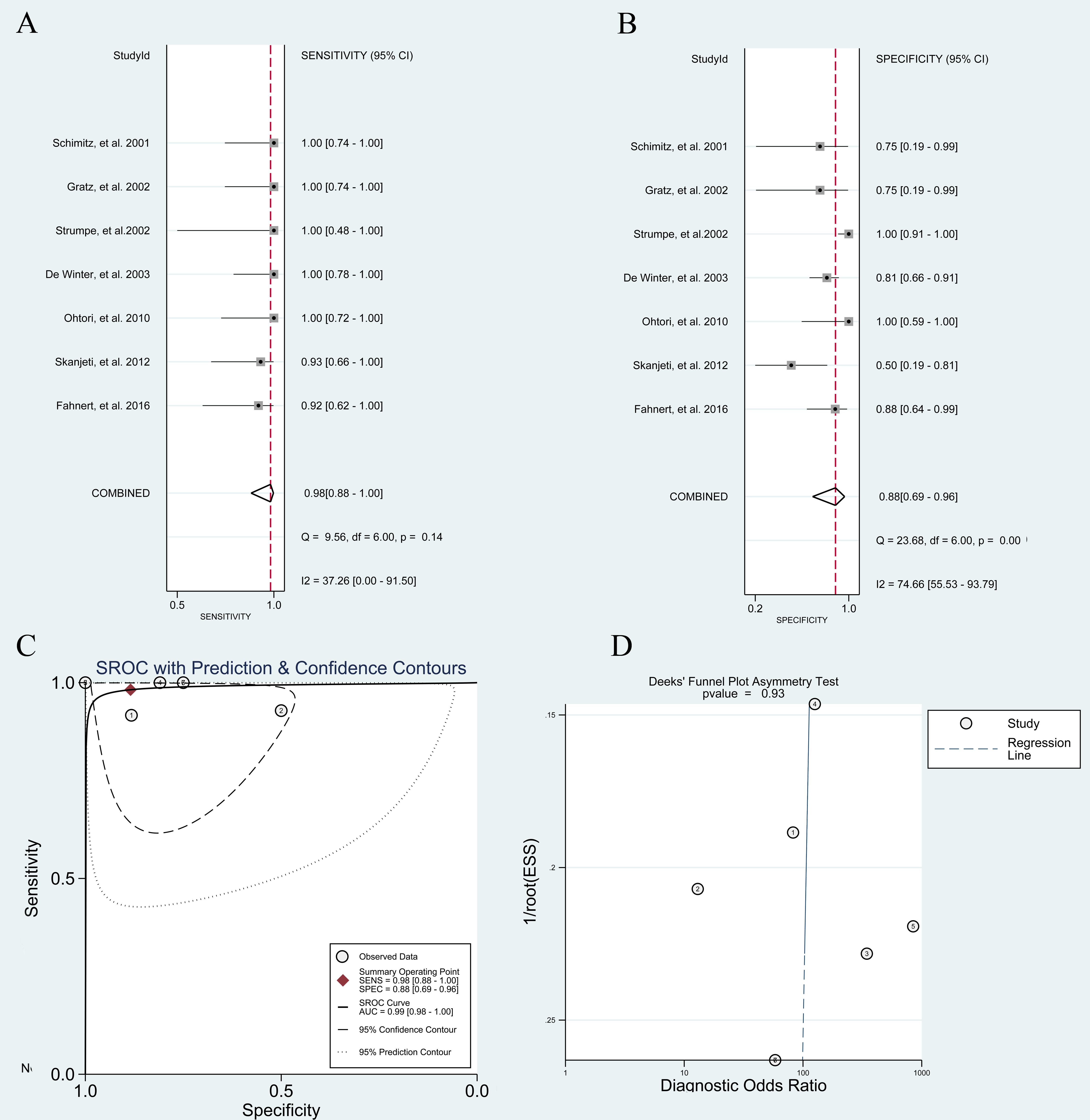

Supplement: Supplementary file 1 — Additional file 1: Fig. S1. Diagnostic performance of 18F-FDG PET for pyogenic spondylitis: (A) pooled sensitivity (B) pooled specificity (C) summary receiver operating characteristic curve (sROC) with the Q*-index and (D) publication bias. [file 13018_2023_3507_MOESM1_ESM.tif]

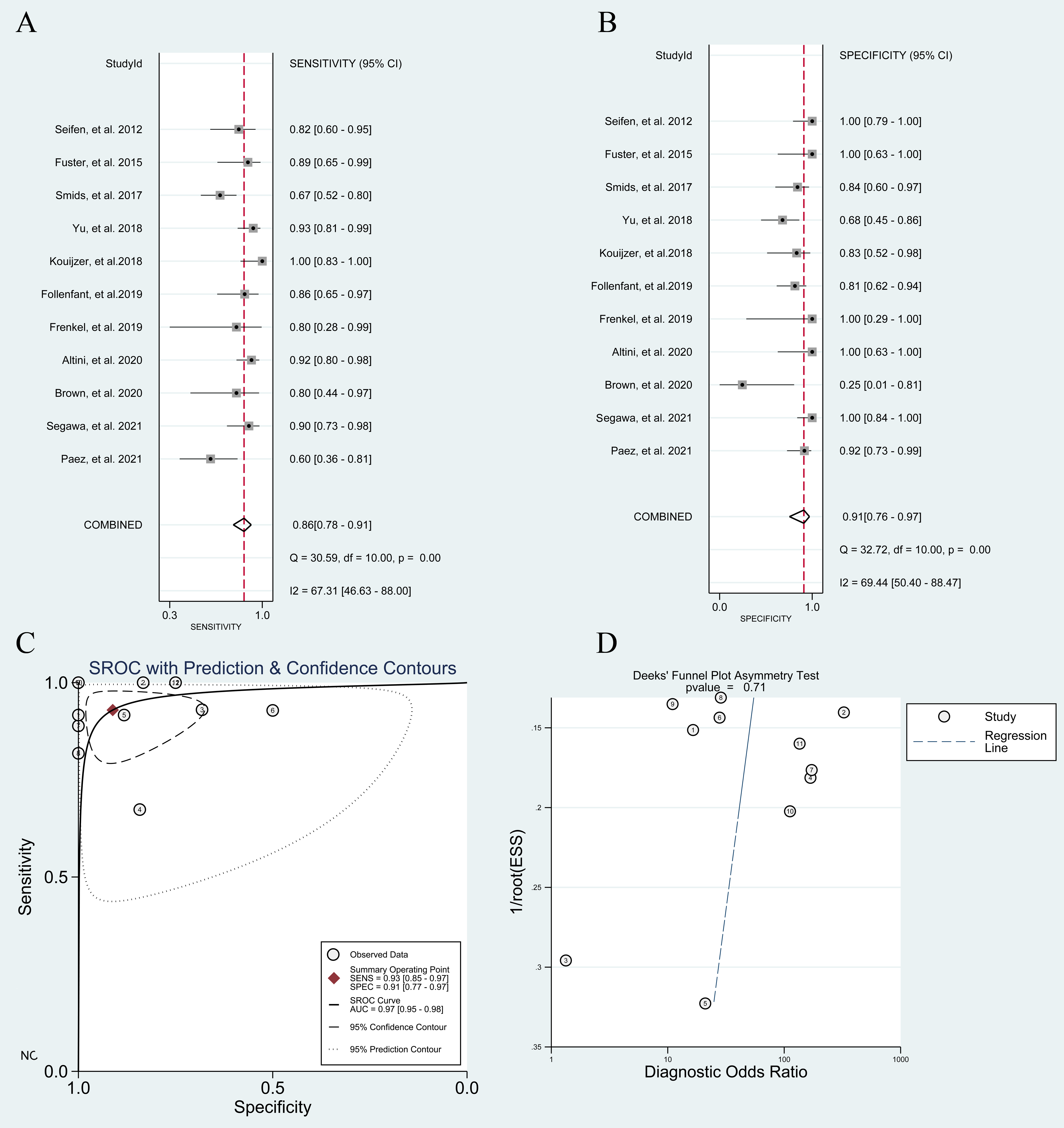

Supplement: Supplementary file 2 — Additional file 2: Fig. S2. Diagnostic performance of 18F-FDG PET/CT for pyogenic spondylitis: (A) pooled sensitivity (B) pooled specificity (C) summary receiver operating characteristic curve (sROC) with the Q*-index and (D) publication bias. [file 13018_2023_3507_MOESM2_ESM.tif]
